# Destructive Clustering of Metal Nanoparticles in Chalcogenide and Oxide Glassy Matrices

**DOI:** 10.1186/s11671-016-1250-y

**Published:** 2016-01-19

**Authors:** M. V. Shpotyuk, O. I. Shpotyuk, J. Cebulski, S. Kozyukhin

**Affiliations:** Lviv Polytechnic National University, Bandera str., 12, Lviv, 79013 Ukraine; Jan Dlugosz University of Czestochowa, al. Armii Krajowej, 13/15, Czestochowa, 42200 Poland; Vlokh Institute of Physical Optics, Dragomanov str., 23, Lviv, 79005 Ukraine; Institute of Materials of SRC “Carat”, Stryjska str., 202, Lviv, 79031 Ukraine; Centre for Innovation and Transfer of Natural Sciences and Engineering Knowledge, Prof. Stanislawa Pigonia str., 1, Rzeszow, 35310 Poland; N.S. Kurnakov Institute of General and Inorganic Chemistry, 31 Leninsky pr., Moscow, 199991 Russia

**Keywords:** Glass, Chalcogenides, Nanoparticle, Host matrix, Chemical bond

## Abstract

The energetic *χ*-criterion is developed to parameterize difference in the origin of high-order optical non-linearity associated with metallic atoms (Cu, Ag, Au) embedded destructively in oxide- and chalcogenide glasses. Within this approach, it is unambiguously proved that covalent-bonded networks of *soft* semiconductor chalcogenides exemplified by binary As(Ge)–S(Se) glasses differ essentially from those typical for *hard* dielectric oxides like vitreous silica by impossibility to accommodate pure agglomerates of metallic nanoparticles. In an excellence according to known experimental data, it is suggested that destructive clustering of nanoparticles is possible in Cu-, Ag-, and Au-ion-implanted dielectric oxide glass media, possessing a strongly negative *χ*-criterion. Some recent speculations trying to ascribe equally this ability to *soft* chalcogenide glasses despite an obvious difference in the corresponding bond dissociation energies have been disclosed and criticized as inconclusive.

## Background

Nanocomposite materials containing functional nanoscale-length inhomogeneities created by embedded metal nanoparticles (MNP) attract a high attention in nowadays materials science community as perspective candidates for advanced sensing application exploring plasmon resonance effects [1–7]. From purely technological standpoint, these nanocomposites are often stabilized through destructive treatment of homogeneous solid-state matrices affected by incorporated MNP, e.g., by producing aggregates (atomic clusters) of dispersed MNP interacting with some unfettered atoms of destroyed substances. The low-energy (tens of keV) ion-implantation technique [6–9] can serve as typical representative example of such destructive nanoclusterization routes. In such a case, just chemical interaction plays a decisive role in the functionality of fabricated nanocomposites defined by MNP concentration, their size, and spatial distribution.

Thus, under implanting copper (Cu^+^), silver (Ag^+^), or gold (Au^+^) ions in *hard* dielectric matrices of vitreous oxides like silica SiO_2_ [10], the *metal-metal* chemical bonding occurs to be more favorable in terms of the Gibbs free energy in respect to competitive *metal-matrix* interaction, resulting in spatially restricted 3–10 nm MNP aggregates [7–9]. However, this is not a case of structurally homogeneous *soft* semiconductor matrices proper to stoichiometric chalcogenides like As_2_S(Se)_3_ or GeS(Se)_2_ [10], where such tiny agglomerates of MNP cannot be stabilized chemically [11].

At the same time, it is well known that highly inhomogeneous chalcohalide 56GeS_2_–24Ga_2_S_3_–20KBr glass due to characteristic inner phase diversity can be well stabilizing media for relatively coarse agglomerates of Ag MNP reaching few hundred nanometers under ion implantation with 10^16^–2⋅10^17^ ions/cm^2^ doses [12, 13]. Noteworthy, in contrast to vitreous oxides, the chalcogenides allow substantial variation in their glass-forming ability without changing in network interlinking in a wide compositional range, possessing spatially homogeneous non-stoichiometric glasses in addition to stoichiometric ones [14, 15]. This feature can be a reason for essentially modified chemical interaction in *metal-matrix* nanocomposites, tuning the conditions for destructive MNP clustering in non-stoichiometric chalcogenide glasses.

In this work, we shall try to parameterize numerically this difference between the expected nanoclustering effects resulting from destructively embedded MNP (Cu, Ag, and Au) in typical oxide SiO_2_-type and chalcogenide As(Ge)-S(Se)-type glass-forming matrices (including compositional non-stoichiometry effects in the latter) from a viewpoint of covalent chemical bond (CCB) approach [16–19].

## Methods

The CCB approach is one of the most efficient semi-empirical tools in the parameterization of chemical interaction in glass-forming systems, which was convincingly exemplified with Bicerano’s and Ovshinsky’s analytical inspection on structures of ovonic-type chalcogenide glasses with reversing electrical-switching properties yet in 1985 [18]. In its basis, this method was justified in the earliest 1970s, the time of fundamental Kastner’s analysis on compositional effects in amorphous semiconductors [17] and pioneering Phillips’s research on chemical bond ionicity in solids [16]. Later, this method gained a wide popularity in the prediction of glass-forming trends in many multicomponent chemical systems, as it was clearly resumed by Feltz in the monograph [14].

Let us consider the specificity of chemical bonding in typical chalcogenide and oxide glass-formers affected by embedded metal dopants from a point of the CCB approach.

The chemical bond distribution in binary (K–X) glass-forming systems, where K denotes cation-type atoms (K=Si, As or Ge) and X stands for anion-type atoms (X=O, S or Se), is governed by thermochemical disproportionality between heteronuclear and homonuclear bonds forming in respect to simple reaction below [14]:

1 $$ 2\left(\mathrm{K}\hbox{--} \mathrm{X}\right)\leftrightarrow \left(\mathrm{K}\hbox{--} \mathrm{K}\right)+\left(\mathrm{X}\hbox{--} \mathrm{X}\right). $$

where notes in brackets define mean molar energy of corresponding bond.

To parameterize different bond-changing structural configurations, we can use mean molar bond energies estimated from standard atomization enthalpies of relevant binary compounds gathered in Table 1 (these energies were determined with ±10 kJ/mol error) [14].

**Table 1:** Mean molar energies *E* (kJ/mol) of chemical bonds in chalcogenide and oxide glasses [14]

Bond	*Е*, kJ/mol	Bond	*Е*, kJ/mol	Bond	*Е*, kJ/mol
As–S	260	As–As	200	Si–O	465
As–Se	230	S–S	280	Si–Si	225
Ge–S	265	Se–Se	225	O–O	250
Ge–Se	225	Ge–Ge	225		

Thus, the energetic balance *∆E* of the above disproportionality reaction Eq. ( 1) is strongly left-shifted in oxide glass-formers such as vitreous silica SiO_2_, approaching 455 kJ/mol. This tendency plays a decisive role in network-forming ability of these glasses, determining full preference of tetrahedral SiO_4/2_ building blocks in their structure. The glass-forming oxides (including also glassy combinations of silica, alkaline and alkaline earth oxides, transition metal oxides and oxides of main group elements like Al_2_O_3_, GeO_2_, B_2_O_3_, P_2_O_5_, TeO_2_, and others) possess high melting and glass transition temperatures, and wide band-gaps proper rather to dielectrics, making them optically transparent and colorless for visible light [14, 15].

In contrast, in chalcogenide glass-formers such as binary As–S(Se) or Ge–S(Se) alloys, the energetic balance *∆E* of thermochemical disproportionality Eq. (1) is much depressed reaching 40–60 kJ/mol only [14]. So, homonuclear bonding occurs to be competitive in respect to heteronuclear bonding in these vitreous systems, this effect being dependent on both glass composition and preparation technology. Appearance of homonuclear bonds was detected even in stoichiometric As_2_S_3_ glasses rapidly quenched from high temperatures more than 800 °C (above boiling point of this compound) [20–23], or, alternatively, cooled from relatively low 450–500 °C temperatures, allowing insufficient mutual solubility of components (As and S) in a melt [24]. These glasses are transparent in IR spectral region up to 15–25 μ, but they possess narrower band-gaps (not exceeding 3 eV, a character limit of semiconductors) [14, 15]. In contrast to vitreous oxides, the chalcogenide glasses possess lower melting and glass transition temperatures. This difference is convincingly evidenced by the fact that vitreous silica ampoules are most suitable crucible materials to prepare chalcogenide glass within melt quenching route [14]. That is why *semiconductor vitreous chalcogenides* are usually termed as *soft* glasses, to be in contrast with *dielectric vitreous oxides* termed as *hard* glasses [10]. The deep reason of such specificity is hidden in an anomalous electronegativity proper to oxygen (O) as compared with relatively mediates values in chalcogenides, thus resulting in different chemistry of their glass-forming networks [15].

Under destructive incorporation of metal M atoms in a glass network (M=Cu, Ag, Au), the existing bond balance can be essentially disturbed owing to chemical interaction with unfettered cation-type K and anion-type X atoms. By analogy with Eq. (1), the corresponding M-bond disproportionality under interaction with X environment solely can be determined as

2 $$ 2\left(\mathrm{M}-\mathrm{X}\right)\leftrightarrow \left(\mathrm{M}-\mathrm{M}\right)+\left(\mathrm{X}-\mathrm{X}\right). $$

If interaction with K environment is more decisive, this reaction attains other form:

3 $$ 2\left(\mathrm{M}-\mathrm{K}\right)\leftrightarrow \left(\mathrm{M}-\mathrm{M}\right)+\left(\mathrm{K}-\mathrm{K}\right). $$

In general, if both types of chemical interaction with X and K environments are equally important for embedded M atoms, the bond disproportionality in (K–X) glass is ruled out by summarizing above Eqs. (2) and (3):

4 $$ 2\left[\left(\mathrm{M}-\mathrm{X}\right)+\left(\mathrm{M}-\mathrm{K}\right)\right]\leftrightarrow 2\left(\mathrm{M}-\mathrm{M}\right)+\left(\mathrm{X}-\mathrm{X}\right)+\left(\mathrm{K}-\mathrm{K}\right)\leftrightarrow 2\left(\mathrm{M}-\mathrm{M}\right)+2\left(\mathrm{K}-\mathrm{X}\right). $$

The left-side part of this Eq. (4) reflects chemical interaction between M and unfettered atoms of destructed glass matrix, while the right-side part describes agglomeration of M atoms in (K–X) matrix renovated after destruction. Therefore, in most generalized case of all interactions possible in M-glass system, the simple difference in molar bond energies

5 $$ {\upchi}^{\mathrm{gen}}=\left[\left(\mathrm{M}-\mathrm{X}\right)+\left(\mathrm{M}-\mathrm{K}\right)\right]-\left[\left(\mathrm{M}-\mathrm{M}\right)+\left(\mathrm{K}-\mathrm{X}\right)\right] $$

can be accepted as a numerical measure describing MNP clustering in a glassy network (clustering is possible under increased negative *χ*^gen^ values).

In realistic *host* systems of glassy oxides and chalcogenides, the unfettered X-type environment of oxygen and chalcogen atoms is undoubtedly most plausible for interaction with *guest* M atoms. So we can ignore (M–K) interactions in the disproportionality balance Eq. ( 4), resulting with respect to Eqs. (1) and (2) in sequent bond transformation:

6 $$ 2\left(\mathrm{K}-\mathrm{X}\right)+\left(\mathrm{M}-\mathrm{M}\right)\leftrightarrow \left(\mathrm{K}-\mathrm{K}\right)+\left(\mathrm{X}-\mathrm{X}\right)+\left(\mathrm{M}-\mathrm{M}\right)\leftrightarrow \left(\mathrm{K}-\mathrm{K}\right)+2\left(\mathrm{M}-\mathrm{X}\right). $$

If energetic barrier *∆E* of this reaction occurs to be positive (the right-shifted equilibrium), the implanted M atoms destroy existing bond distribution in a *host* glass due to heteronuclear (M–X) bonds formed at cost of homonuclear (K–K) ones. Otherwise, the MNP agglomeration occurs owing to the preference of (M–M) interaction and renovation of destructed (K–X) bonds (the left-shifted equilibrium).

Thereby, we can introduce the energetic *χ*-criterion to justify clustering of *guest* MNP embedded destructively in *host* stoichiometric (K–X) glassy matrix:

7 $$ {\upchi}^{\mathrm{st}}=2\left(\mathrm{M}\hbox{--} \mathrm{X}\right)+\left(\mathrm{K}\hbox{--} \mathrm{K}\right)\hbox{--} 2\left(\mathrm{K}\hbox{--} \mathrm{X}\right)\hbox{--} \left(\mathrm{M}\hbox{--} \mathrm{M}\right)=2\left[\left(\mathrm{M}\hbox{--} \mathrm{X}\right)\hbox{--} \left(\mathrm{K}\hbox{--} \mathrm{X}\right)\right]+\left[\left(\mathrm{K}\hbox{--} \mathrm{K}\right)\hbox{--} \left(\mathrm{M}\hbox{--} \mathrm{M}\right)\right]. $$

Mean molar energies of (M–X) bonds for different metal atoms M (M=Cu, Ag or Au) in oxide and chalcogenide environment are given in Table 2 [25]. Under a comparison with data of Table 1, it is evident that these bond energies are strongly reduced as those of Si–O bonds, but they are comparable and even greater in respect to the corresponding energies of heteronuclear bonds in chalcogenide glasses. It means that under M ion implantation, the destructed heteronuclear Si–O bonds in SiO_2_ glass are renewed, facilitating agglomeration of “pure” MNP in *host* bulk glass (provided ion implantation dose is sufficient to ensure MNP excess above solubility limit [6–9]). Hence, the energetic barrier *χ*^st^ defined by Eq. (7) is negative for oxide glasses. But this situation is changed in case of chalcogenide environment, where *χ*^st^ value is positive for embedded Cu atoms and one-order smaller as in vitreous oxides. For Ag and Au atoms in As–S or Ge–S environment, this barrier becomes negative, but further not exceeding a few tens of kJ/mol.

**Table 2:** Mean molar bond energies *E* (kJ/mol) for different metallic atoms in oxide and chalcogenide environment [25]

Bond	*Е*, kJ/mol	Bond	*Е*, kJ/mol	Bond	*Е*, kJ/mol
Cu–Cu	200	Ag–Ag	165	Au–Au	225
Cu–O	290	Ag–O	220	Au–O	225
Cu–S	275	Ag–S	220	Au–S	255
Cu–Se	255	Ag–Se	210	Au–Se	250

In case of non-stoichiometric chalcogenide glass-forming systems, the above Eq. (6) should be considered separately for intermetallic (M–M) bonding in heteronuclear (K–X) and homonuclear (K–K) and (X–X) environments, the corresponding bond disproportionality reactions being as follows:

8 $$ 2\left(\mathrm{K}\hbox{--} \mathrm{X}\right)+\left(\mathrm{M}\hbox{--} \mathrm{M}\right)\leftrightarrow 2\left(\mathrm{M}\hbox{--} \mathrm{X}\right)+\left(\mathrm{K}\hbox{--} \mathrm{K}\right), $$

9 $$ 2\left(\mathrm{X}\hbox{--} \mathrm{X}\right)+\left(\mathrm{M}\hbox{--} \mathrm{M}\right)\leftrightarrow 2\left(\mathrm{M}\hbox{--} \mathrm{X}\right)+\left(\mathrm{X}\hbox{--} \mathrm{X}\right), $$

10 $$ 2\left(\mathrm{K}\hbox{--} \mathrm{K}\right)+\left(\mathrm{M}\hbox{--} \mathrm{M}\right)\leftrightarrow 2\left(\mathrm{M}\hbox{--} \mathrm{K}\right)+\left(\mathrm{K}\hbox{--} \mathrm{K}\right). $$

The energetic preference of resulting bond balance in a glass *∆E* can be estimated by accepting weighting coefficients *η* of different bonds possible under a given structural model:

11 $$ \begin{array}{l}{\upeta}_{\mathrm{K}\hbox{--} \mathrm{X}}\times \left[2\left(\mathrm{K}\hbox{--} \mathrm{X}\right)+\left(\mathrm{M}\hbox{--} \mathrm{M}\right)\right]+{\upeta}_{\mathrm{X}\hbox{--} \mathrm{X}}\times \left[2\left(\mathrm{X}\hbox{--} \mathrm{X}\right)+\left(\mathrm{M}\hbox{--} \mathrm{M}\right)\right]+{\upeta}_{\mathrm{K}\hbox{--} \mathrm{K}}\times \left[2\left(\mathrm{K}\hbox{--} \mathrm{K}\right)+\left(\mathrm{M}\hbox{--} \mathrm{M}\right)\right]\leftrightarrow \\ {}\leftrightarrow {\upeta}_{\mathrm{K}\hbox{--} \mathrm{X}}\times \left[2\left(\mathrm{M}\hbox{--} \mathrm{X}\right)+\left(\mathrm{K}\hbox{--} \mathrm{K}\right)\right]+{\upeta}_{\mathrm{X}\hbox{--} \mathrm{X}}\times \left[2\left(\mathrm{M}\hbox{--} \mathrm{X}\right)+\left(\mathrm{X}\hbox{--} \mathrm{X}\right)\right]+{\upeta}_{\mathrm{K}\hbox{--} \mathrm{K}}\times \left[2\left(\mathrm{M}\hbox{--} \mathrm{K}\right)+\left(\mathrm{K}\hbox{--} \mathrm{K}\right)\right].\end{array} $$

where the left side reflects energetic balance of agglomerated MNP within renewed *host* matrix, and the right side corresponds to M atoms interacting with unfettered atoms of destructed glass.

In realistic non-stoichiometric chalcogenide glassy media, the chemical interaction between embedded metallic M and cation-type K atoms can be ignored [14], thus resulting in importance of only two first components in both the left and right sides of the above Eq. (11) to calculate the energetic *χ*-criterion in non-stoichiometric chalcogenide glass matrices:

12 $$ \begin{array}{l}{\upchi}^{\mathrm{nst}}={\upeta}_{\mathrm{K}\hbox{--} \mathrm{X}}\times \left[2\left(\mathrm{M}\hbox{--} \mathrm{X}\right)+\left(\mathrm{K}\hbox{--} \mathrm{K}\right)\right]+{\upeta}_{\mathrm{X}\hbox{-} \mathrm{X}}\times \left[2\left(\mathrm{M}\hbox{--} \mathrm{X}\right)+\left(\mathrm{X}\hbox{--} \mathrm{X}\right)\right]\hbox{--} \\ {}\hbox{--} {\upeta}_{\mathrm{K}\hbox{--} \mathrm{X}}\times \left[2\left(\mathrm{K}\hbox{--} \mathrm{X}\right)+\left(\mathrm{M}\hbox{--} \mathrm{M}\right)\right]\hbox{--} {\upeta}_{\mathrm{X}\hbox{--} \mathrm{X}}\times \left[2\left(\mathrm{X}\hbox{--} \mathrm{X}\right)+\left(\mathrm{M}\hbox{--} \mathrm{M}\right)\right] = \\ {}={\upeta}_{\mathrm{K}\hbox{--} \mathrm{X}}\times \left[2\left(\mathrm{M}\hbox{--} \mathrm{X}\right)\hbox{--} 2\left(\mathrm{K}\hbox{--} \mathrm{X}\right)+\left(\mathrm{K}\hbox{--} \mathrm{K}\right)\hbox{--} \left(\mathrm{M}\hbox{--} \mathrm{M}\right)\right]+{\upeta}_{\mathrm{X}\hbox{--} \mathrm{X}}\times \left[2\left(\mathrm{M}\hbox{--} \mathrm{X}\right)\hbox{--} \left(\mathrm{X}\hbox{--} \mathrm{X}\right)\hbox{--} \left(\mathrm{M}\hbox{--} \mathrm{M}\right)\right].\end{array} $$

## Results and Discussion

Let us apply the above energetic parameterization route to describe the MNP clustering in non-stoichiometric and defective chalcogenide matrices.

**Fig. 1:**
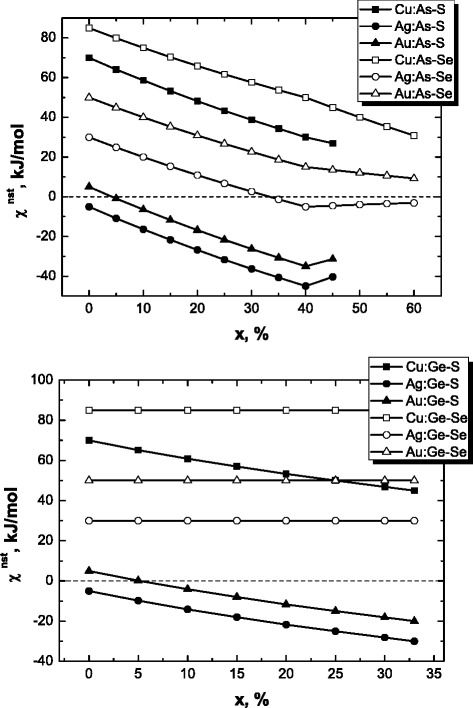
Compositional dependences of energetic *χ*-criterion describing destructive clustering of MNP (Cu, Au, and Ag) in As_*x*_S(Se)_100-*x*_ (*top*) and Ge_*x*_S(Se)_100-*x*_ glasses (*bottom*)

By accepting mean molar bond energies gathered in Tables 1 and 2 for As(Ge)_*x*_S(Se)_100-*x*_ glasses and weighting coefficients *η* determined within chemically ordered covalent network model preferring heteronuclear bonding [14, 26, 27], the energetic *χ*-criterion for MNP clustering in respect to Eq. (12) can be parameterized as shown in Fig. 1. It is obvious that non-stoichiometry has no significant effect on *χ*^nst^ value in these glasses, the over-stoichiometric chalcogen atoms only enhancing interaction with metallic M atoms significantly preventing MNP clustering. This non-clustering tendency is better revealed in case of Cu atoms embedded in selenide glass environment, while Ag atoms in sulfide glasses mainly assist in an inverse trend.

Noteworthy, the *χ*-criterion describing MNP clustering in dielectric oxide environment of vitreous silica SiO_2_ essentially differs as compared with those proper to semiconductor vitreous chalcogenides like As(Ge)_*x*_S(Se)_100-*x*_ (see Fig. 1), respectively, approaching −325, −430, and −480 kJ/mol for Cu, Ag, and Au atoms. So, Au is the most appropriated candidate to be agglomerated in the destructed environment of heteronuclear Si–O chemical bonds, while Cu demonstrates relatively weaker tendency. This remarkable ability of embedded M atoms predicted via large negative values of *χ*-criterion is in excellent harmony with known results in successful fabrication of compactly aggregated MNP with character 3–10 nm sizes in transparent oxide glass matrices exploring low-energy ion implantation technique [7–9].

At the other hand, from position of the developed approach, it is simply evident misleading the character of recent speculations by some authors [28] ascribing MNP clustering ability equally to typical chalcogenide and oxide glasses. They claimed, in part, that spherical agglomerates of pure Cu MNP having only 5–10 nm in sizes can be fabricated by low-energy (40 keV) Cu^+^-ion implantation in As_2_S_3_ glass, like it occurs in vitreous silica SiO_2_. However, from a standpoint of scientific authenticity, none of the arguments given in [28] testify in a favor of this conclusion. Thus, (1) non-linear Z-scan pattering was performed only for ion-implanted glass without any reference to its initial state, (2) characteristic plasmon resonance band expected from Cu MNP in high-refractive environment (*n* ≈ 2.5 for As_2_S_3_ glass [1, 14, 15]) was positioned at ~580–590 nm, e.g., at the same wavelengths as in low-refractive (*n* < 2.0) oxide glass environment, (3) optical transmission spectrum of 1-mm thick As_2_S_3_ glass was surprisingly not affected by implantation even at such high ion-beam doses as 10^17^ cm^-2^ (excepting 580–590 nm range), but unrealistically shifted towards shorter wavelengths (nearly ~500 nm) and sharply suppressed in transmittance (not character for *n* ≈ 2.5). These circumstances apparently testify that results of Kavetskyy et al. [28] are clearly meaningless. The unreasonably announced effect of small-sized Cu MNP ion-synthesized identically in chalcogenide and oxide glasses was obviously falsified in [28], despite further attempts of these authors to wimple this by comparison with well-approbated non-linear optical effects in polarizing oxide glasses with silver nanorods [29].

One of the most effective ways to overcome the above restrictions of bond-redistribution schemes activated in *host-guest* systems is to explore nanostructurization under *non-disturbed bond balance* in a *host* glassy matrix. This resolution can be well illustrated by glass deposition in MNP initially formed at a surface of dielectric substrate, when upper film plays only a rope of covering layer ensuring necessary difference in optical refractive index *n* with these MNPs [11, 30–32]. M-glass interaction in such system is essentially suppressed because of the absence of unfettered atoms supplied from covering glass layer.

Other example concerns the case, when regular chemical interaction in *host-quest* system is inhibited due to inhomogeneous structure of some glass targets. This resolution can be well exemplified by multicomponent chalcohalide glasses and ceramics, where structural polyhedrons of typical chalcogenide networks are composed with ionic halide groups [12, 13]. Thus, in 56GeS_2_–24Ga_2_S_3_–20KBr glass, the Ag MNP embedded under ion implantation with varied doses from 10^16^ to 2 ⋅ 10^17^ ions/cm^2^ can be gathered presumably in the inner spaces of lower atomic densities, which allows appearance of relatively large MNP agglomerates (few hundred of nm). The enhanced third-order optical non-linearities in these nanostructured chalcohalide glasses correlate well with ion implantation doses and sizes of MNP clusters.

## Conclusions

Different origin of high-order optical non-linearity associated with MNP embedded destructively in oxide- and chalcogenide glassy environment is evidenced from CCB approach, describing this effect in terms of mean molar bond energies character for inner chemical interaction between unfettered components of *host* glassy network and *guest* metal atoms (Cu, Ag, Au). The energetic barrier in the disproportionality balance of all possible chemical interactions in metal-glass system can be accepted as numerical *χ*-criterion parameterizing this difference. Agglomeration of MNP is proper to nanocomposites based on oxide glasses like vitreous silica with strong negative *χ*-criterion (−300 ÷ −500 kJ/mol). In contrast, the MNP clustering is inhibited in chalcogenide glasses by an opposite trend in preferential interaction between embedded metal and destructed glass components in respect to much smaller *χ*-criterion (tens of kJ/mol). Chemical non-stoichiometry has no significant effect on *χ*-criterion in these glasses, since over-stoichiometric chalcogen only enhances interaction with embedded metal atoms significantly preventing their clustering. These findings are in an excellent agreement with numerous evidences exploring destructive routines of metal-glass nanostructurization, but contradict to speculations with unproved embedding schemes for MNP in vitreous chalcogenides like glassy As_2_S_3_.

## Abbreviations

K: cation-type atom
M: metal atom
MNP: metal nanoparticles
X: anion-type atom
